# Experience of emergency medicine residents toward an implemented modified teaching approach

**DOI:** 10.3389/fmed.2023.1152892

**Published:** 2023-09-18

**Authors:** Kholoud Abdullah Babkair, Sami Al-Nasser, Abdullah Alzahem

**Affiliations:** ^1^College of Medicine, King Saud bin Abdulaziz University for Health Sciences, Jeddah, Saudi Arabia; ^2^King Abdulaziz Medical City, Jeddah, Saudi Arabia; ^3^Ministry of National Guard Health Affairs, Jeddah, Saudi Arabia; ^4^Department of Medical Education, College of Medicine, King Saud bin Abdulaziz University for Health Sciences, Riyadh, Saudi Arabia; ^5^King Abdullah International Medical Research Center (KAIMRC), Riyadh, Saudi Arabia; ^6^Ministry of National Guard Health Affairs, Riyadh, Saudi Arabia

**Keywords:** emergency medicine, residency education, active learning, group based learning, inquiry-based learning, small group discussion

## Abstract

Lecturing has always been one of the traditional instructional methods in medical education. It is cost-effective, especially when it comes to conveying a large amount of information to many students at once. However, disadvantages are plenteous, one of which is its passive way of knowledge delivery and learning. Active learning, on the contrary, has better students' engagement and longer retention, and it results in better students' achievement. The emergency medicine residency training program at KAMC-Jeddah has modified the educational activity to become more aligned with the end-of-year assessment in the form of active learning. This study aimed to explore the experience of the residents regarding the implementation of the new educational approach. An exploratory-qualitative study utilizing constructive grounded theory was conducted, collecting our data through an in-depth 1:1 interview using semi-structured open-ended questions. Purposeful sampling was used, and saturation was reached after interviewing 24 residents. The general perception of residents toward the new teaching modes slightly varied, highlighting the positivity of the new educational environment, the desired impact on their learning, the challenges they encountered, and finally their high satisfaction level and support for this new experience. It was asserted that such experience could be permanently implemented to increase the efficacy of teaching and learning.

## Introduction

Lecturing has always been one of the traditional instructional methods in medical education (1). It is defined as teaching by the spoken word, with particular emphasis on the student listening and the teacher talking (2). Lecturing has also been a cost-effective method for conveying large amounts of information to many students (3). Moreover, it is a helpful method for providing explanations in medicine (4).

However, disadvantages are plenteous, especially if learners have different levels, backgrounds, and thought paces. Furthermore, it becomes increasingly difficult to maintain attention when students passively learn, seldom interact with teachers, and need feedback on their questions or thoughts (2).

On the other hand, engaging the students in the learning process is considered active learning (5). In a systematic review and meta-analysis study that compared outcomes from traditional lecture-based learning vs. active learning, more than 225 studies were reviewed, and it was found that students participating in active learning had 6% better exam results than traditional learning students. They were also 1.5 times less likely to fail (1).

One of the active learning strategies widely used is collaborative learning. Collaborative learning requires learners to engage in groups rather than work individually, whether solving a problem, completing a task, or creating a product (6). Over the decades, researchers have studied the benefits of collaborative learning regarding outcomes. Improved academic achievement, self-esteem, and interpersonal interaction were reported in a review article that was updated 6 years later (7, 8). Moreover, it improved students' attitudes and knowledge retention in academic programs (9).

In particular, small group discussion (SGD) is an example of collaborative learning. Several studies have shown its positive effect on learning (9–11). As O'Donnell described, there are mainly two main theories behind its effectiveness, namely, a cognitive theory and a socio-behavioral theory. Cognitively, SGD enhances the elaboration of the topic on a deeper level to prolong knowledge retention. On the socio-behavioral level, SGD enhances motivation and interpersonal connection and helps create positive socio-cultural values (12). Several years later, the cognitive theory behind SGD was further explored experimentally when Van Blankestien found no difference in recall between the collaborative learning group and the control group. However, the recall was better in the experimental group 1 month later, which justifies that discussion positively affects long-term memory (13).

Other examples of collaborative learning are flipped classrooms and case-based discussions. In a literature review article on flipped classrooms, Deng (14) defines *flipped classrooms* as “a pedagogical method in which students learn new knowledge through short videos, podcasts, e-books as well as internet outside class and consolidate what they acquired through classroom activities with the help of classmates and teachers.” On the other hand, case-based learning (CBL) is widely used in undergraduate and postgraduate medical training residency programs (15). The benefits of both strategies mentioned above include connecting theory to practice, stimulating deeper learning, and fostering residents' exploration, evaluation, and reflection (16, 17). In a small study conducted at the Internal Medicine Department at Hamad University, State of Qatar, 64% of internal medicine residents preferred CBL over traditional lectures, 87% concluded that the CBL teaching strategy improved their knowledge, and 84% approved its interactive nature over lectures (18).

Inquiry-based learning (IBL) has been used in education since the times of Socrates (468-399 B.C.), Plato (427-347 B.C.), and Aristotle (384-322 B.C.). According to Barrow (19), IBL helps develop a deeper understanding by nurturing students' critical thinking and scientific reasoning (19). Moreover, it is a valuable method to enhance collaboration, communication, deep thinking, and creativity (20).

Since its establishment, the Emergency Medicine Residency Training Program in the western region of Saudi Arabia has been conducting its full-day educational activity once per week using traditional lecture-based learning to deliver the theoretical component of the curriculum. As the program started to grow, the disadvantages of this teaching strategy became evident, and residents' engagement could have been much better. Such difficulties included a lack of motivation to attend, less interaction with the presenter, less engagement in the topic, and, therefore, less learning. At King Abdulaziz Medical City-Jeddah (KAMC-J), the Emergency Medicine Residency Training Program Committee has incorporated active learning into the weekly educational day to overcome such learning obstacles.

As part of ensuring the quality of the emergency medicine residency program at KAMC-Jeddah, this study aimed to explore the residents' experiences regarding implementing the new teaching and learning approach.

Objectives:

To explore the experience of residents about the implementation of the new active teaching strategies in terms of:

° Engagement
° Positive and negative impact
° Obstacles

## Methodology

### Design

The study took the form of an exploratory qualitative design. Data were collected using semi-structured, in-depth interviews with the emergency medicine residents at King Abdulaziz Medical City-Jeddah. The resident's experience with the modified teaching was studied using a constructive grounded theory approach.

### Study setting

The study was conducted at King Abdulaziz Medical City in Jeddah. Currently, the institute has 33 residency and 23 fellowship training programs. The total number of trainees is 567. The Emergency Medicine Residency program was established in 2002 as the first emergency medicine program in the western region. In the following years, multiple programs were established and joined the mother program, constituting the joint emergency medicine program in the western region. The emergency medicine program at KAMC-J became a separate program in 2020. Complete programs earn accreditation based on specific criteria proposed and followed by the SCFHS in Saudi Arabia. The Emergency Medicine program at KAMC-J can accept 10 new residents per year. The educational activity was separated from the regional program activity in October 2022. At the time of conducting this study, the program had 36 residents. Twenty-six residents had experienced lecture-based teaching, whereas ten were new to the program.

The modified learning approach was implemented for a whole academic year (October 2021 to June 2022). The educational activity schedule introduced the theoretical part in the morning. The residents were divided into eight small groups, with a minimum of three residents and a maximum of six in each group. Each group had a ratio of one senior to two junior residents. Members were attached to their assigned groups for the whole academic year. The didactic lecture was then dissected into chunks, giving the residents enough time to discuss each chunk, followed by questions and answering techniques. Questions were designed following the objectives of the Saudi Commission for Health Specialties (SCFHS) emergency medicine curriculum. Residents were allowed to search for information and introduce updated knowledge to the topic. Based on previous years' residents' recommendations, the afternoon periods also included practical sessions. Those practical sessions included simulation, structured oral exam (SOE), and multiple-choice questions (MCQs) revision sessions. Three hands-on workshops were conducted to familiarize the residents with high-acuity emergency medicine procedures and to train them on the skills they need to maintain. The aim of introducing the above methods was to enhance the residents' clinical experience and to align the teaching methods with the final-year assessment strategy, which included both MCQs and SOE.

### Study population and sampling technique

The study targeted all residents of the Emergency Residency Training Program at KAMC-Jeddah in the academic year 2021–2022. The sampling technique used to select the participants was purposeful sampling. In-depth interviews took place between 12 October 2022 and 4 November 2022 once the Institutional Review Board (IRB) approved the research proposal at the King Abdullah International Medical Research Centre (KAIMRC).

Both IRB approval and consent forms were presented to the residents in advance. Interviews were conducted using the Zoom Video Program. The residents consented to the video recording of the interview, which was later used for thematic analysis.

In total, 12 male and 12 female residents were interviewed, of whom 13 were seniors (in their third and fourth years) and 11 were juniors (in their first or second year). The selection of residents was prioritized based on their own free time. In all, six residents were newcomers. Subject characteristics are explained in Table 1.

**Table 1:** Demographic characteristics of the study subjects.

**Subjects characteristics**	**Number**	**Percentage**
**Gender**		
Male	12	50
Female	12	50
**Level** ^*^		
R1	6	25
R2	5	21
R3	8	33
R4	5	21
Total seniors	13	54
Total juniors	11	46
Total subjects	24	100

^*^Residency level is shortened to R.

### Data collection and management

Data were collected using semi-structured, open-ended interview questions refined and adjusted after meeting with the Educational Activity Committee members based on the aims and objectives of this study. The meeting aimed to pilot the questions and brainstorm for any additional ones to probe the residents' experience.

The interview guide contained five domains: demographic data, satisfaction experience, advantages and disadvantages of the modified teaching approach, challenges experienced, and recommendations for improvement. All interviews were conducted in English and videotaped. Recordings were transcribed verbatim manually and then imported into the data analysis program.

### Data analysis

NVIVO (QSR International Pty Ltd.) version 20.7.0 was used for data analysis. The thematic analysis was performed according to the six phases proposed by Braun and Clarke (21). Data were transcribed verbatim manually and then imported to the NVIVO after being checked against the recording for accuracy. The text was read and re-read to identify possible codes across the data. Each data item was given equal attention in the coding process. The coding process was comprehensive and conclusive, aiming to generate the appropriate themes. The themes were reviewed and revised multiple times by the research team. Themes and attached references were then exported to a Word document to generate the final report.

### Findings

The participants were divided into senior and junior levels. In total, 12 male and 12 female residents were interviewed, of whom 13 residents were seniors (in their third and fourth year) and 11 were juniors (in their first or second year). Saturation was reached by the 24th resident. The average length of the interview was between 40 and 70 min.

**Figure 1:**
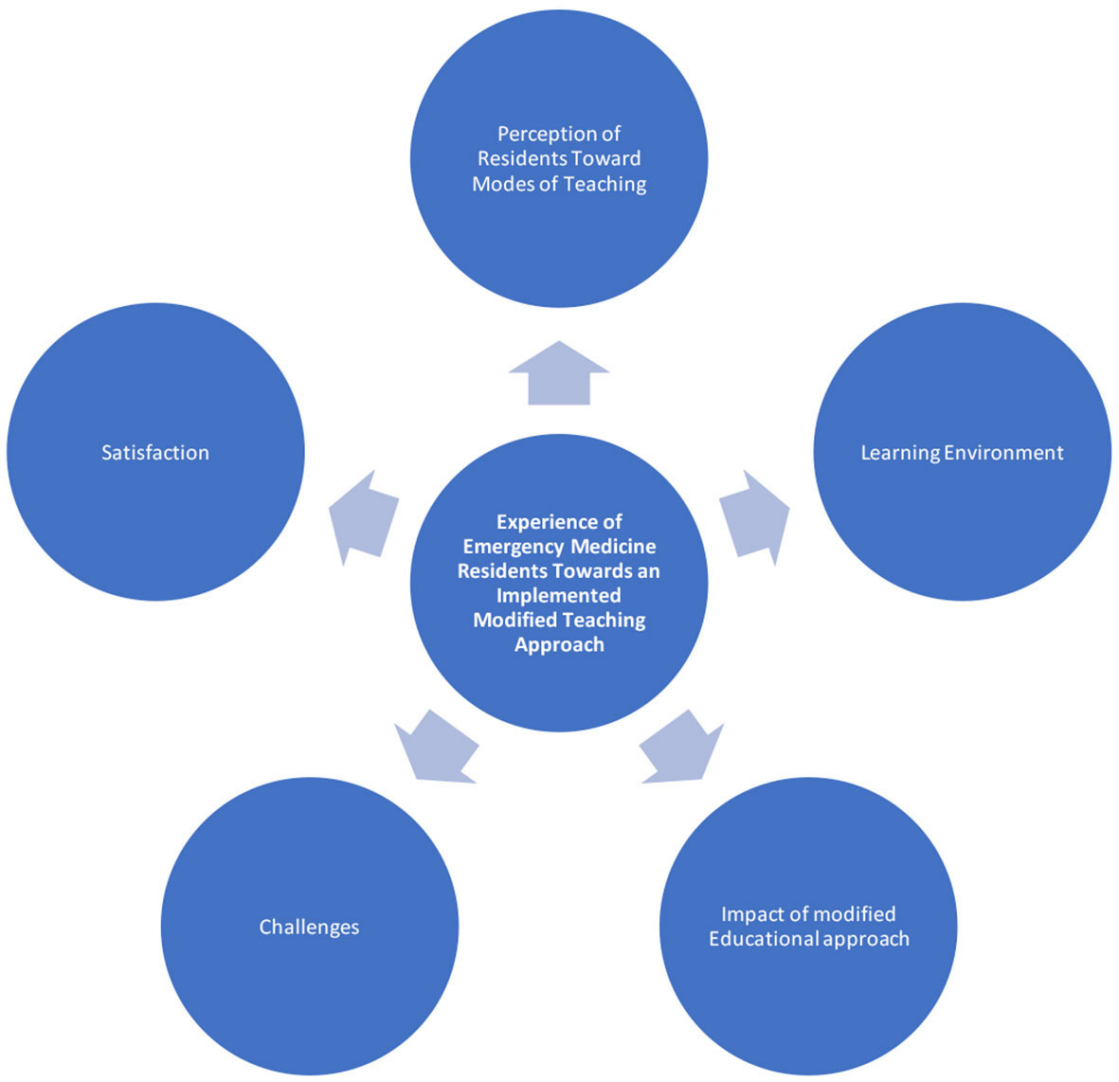
Illustrative thematic analysis map.

Five main themes were identified and explored and are believed to thoroughly explain the residents' experience with the new educational approach. The main themes generated from our data were the general perception of residents toward the new teaching modes, the learning environment, the impact of the new educational approach, the challenges they encountered, and their general satisfaction level with the new experience. The thematic analysis map is displayed in Figure 1. Table 2 shows an example of referenced codes and themes.

**Table 2:** Examples of referenced codes and themes.

**Name**	**Files**	**References**
1-Mode of teaching	0	0
1-Small group discussion	5	11
Advantage of group discussion	3	5
Development of skills for group interactions	11	17
Effect on home duty	3	7
Effect on learning	4	11
Correction of the misconception	5	8
Engagement and Peer teaching	7	9
Improved knowledge retention	3	3
Improved reiteration of knowledge	2	2
Improved knowledge compression	7	8
Meaningful learning experience	1	1
Disadvantages of the small group discussion	3	5
Cognitive overload	1	1
Effect of reading the topic before the discussion	3	6

#### Theme 1: General perceptions of residents toward the new teaching modes

There are three different teaching modes the residents were exposed to:

1. They experienced the theoretical knowledge delivery mode, which consisted of small group discussions and monthly MCQ sessions.
2. They went through a practical mode that consisted of monthly simulation sessions, biweekly SOE sessions, and hands-on workshops.
3. They took part in both physical and online teaching modes.

##### Sub-theme 1: Theoretical knowledge delivery

The residents felt more engaged in the educational cycle; they were constructing new knowledge, solidifying the existing knowledge, and having their misconceptions corrected. They believed the formerly mentioned benefits had improved knowledge retention and reiteration much better than traditional lectures. Moreover, small group discussions made them more enthusiastic about learning, resulting in a more meaningful learning experience.

*Small group discussion made me interested to learn the topics that I have to know about. It made the learning experience more interesting and thus made the learning more meaningful. (J3)*

The majority felt that the new approach also helped them know each other better, share their knowledge freely, irrespective of their level, develop rapport toward each other, and respect each other's opinions. Such skills are essential when it comes to group discussion.

*I had the opportunity to share my knowledge with the group which built my enthusiasm to share knowledge again. (J3)*

*One of the advantages is establishing rapport with colleagues. (S4)*

Moreover, seniors believed they had an opportunity to develop mentoring and coaching skills for the juniors, whereas juniors believed that senior contribution to the educational cycle was a significant advantage.

*Small Group Discussion helps mentoring and guiding the junior in the group. (S4)*

*It increased the level of understanding as a junior, and I was discussing at my level, and I had additional feedback from the R3 and R4. (J10)*

Another advantage of small group discussions over traditional lectures is the better preparation for the final exam and the effect on home study. They believed the question-and-answer technique in the small group discussion helped point out the preliminary information. This technique improved their home study because they had to pre-read the topics to contribute to the discussion. They also better understood the topic when they studied it again after the activity. However, they described the small group discussion as useless when group members did not read beforehand and, accordingly, had less collaboration. Another disadvantage was the cognitive overload they felt toward the end of the activity and the amount of mental exhaustion. They argued that they had to stay attentive all the time compared to the traditional lectures, where they could blank out when needed as their continuous participation was not necessary. Although this was discussed as a disadvantage, it explains why this activity helped improve learning as it caught their attention all the time.

*I need to be all the time attentive which sometimes made me cognitively overloaded. (S5)*

Other disadvantages included the need for more training in professional presentation skills for juniors. Some seniors believed that the new method did not cultivate the proper presentation skills juniors needed for future professional talks at conferences, and that was a drawback seen by seniors.

*No practice of solo presentation skills so they can't develop presentation skills to huge audience. (S2)[SIC]*

Regarding the disadvantages related to the groups themselves, issues such as being assigned to one group the whole year, the random assignment of the members to the group, and the effect of the absence of the group members on the quality of the discussion were raised. They also described the new technique as time-consuming compared to the traditional lectures in terms of preparation and conduct. However, they described the lectures as an ineffective tool to add to their learning as they lack discussion, do not grasp their attention long enough, and eventually do not result in information retention.

*You can't guarantee learning is happening. (J5)*

*The information retaining is nearly zero. (S5*)

Boredom, lack of interaction, and lack of interest in studying were also mentioned as disadvantages of traditional lectures. However, only two residents mentioned that lectures provided them with more comprehensive review material that helped their reading. One resident mentioned she used to have better comprehension and retention of the topic when she prepared for lectures, as she was covering more information than just the objectives.

When asked about their perception of the monthly MCQ sessions, the majority regarded the sessions as a challenging and effective tool to keep them studying, putting them in the hot zone of sitting for the exams and providing feedback that helped them know their level.

*The MCQ revision at the end of the month have given me a lot of advantage to know your exact level and to keep you in the mood of exam all the time. Gave me more confidence. (S6)[SIC]*

##### Sub-theme 2: Practical knowledge delivery

Additional modes of teaching were practical teaching sessions, including simulation, SOE preparation sessions, and hands-on workshops. The majority found the simulation beneficial and impacted their practice in learning and maintaining their leadership skills in managing sick patients, such as delegating, prioritizing actions, sharing information, decision-making, coordinating, coaching team members if needed, and closed-loop communication skills.

*Simulation experience give a quality time teaching. (J3)[SIC]*

However, one of the senior residents had difficulty appreciating its effectiveness since she was already mentally exhausted after a long day. Another senior resident felt cases were constantly challenging, which caused some frustration when he failed to act as needed.

*Simulation did not add to me at any point. I felt tired all the times by the time simulation is over I had no energy and I just wanted to go home. (S7)[SIC]*

On the other hand, the biweekly SOE preparation sessions gained the approval of all juniors and seniors as an effective tool to review and get oriented for the final SOE.

*Oral cases prepared us to the SOE well with good advantages, common cases, and challenging cases. The providers were well-oriented to the new way of conducting SOE. (J3)[SIC]*

Regarding the hands-on workshops, all residents believed that workshops were good tools for maintaining practical skills, especially for procedures not frequently encountered in actual practice.

##### Sub-theme 3: Physical vs. online teaching

The residents had to experience both modes, physical attendance and online, in the years 2021–2022 due to the institutional social restrictions of COVID-19 that were applied for a few months. We assimilated the physical group distribution with the breakout room option in online mode using the Zoom program. Toward the end of the academic year and the resolution of the restrictions, the EAC decided to incorporate both modes into one hybrid mode, where the residents attended physically for the first 2 weeks of the month and then online for the remaining 2 weeks.

Most residents preferred the physical attendance (onsite in the residents' quotes) to the online mode, arguing that it helped their engagement and interaction. Moreover, they highly regarded the social aspect of the experience, where they could meet and greet their friends and spend quality time. Some residents also mentioned better group management when attending physically, better focus, better collaboration, and more senior-to-junior observation.

*Onsite is better, more focused, and more attendance because you can't guarantee the presence of the students if camera is off. (S3)*

*Onsite: Face to face, I can see all, I can manage my group better. Better group interaction although the commute takes long hours. (J9)*

While most residents voted for physical attendance, some of them favored the online mode that allowed them to save the commute time to and from the hospital. Moreover, they considered the online time as break time, which they attended comfortably from their place of stay. However, many disregarded the online mode as it was easy to disengage mentally, and some had technical difficulties.

*Online was more comfortable but not beneficial. (S3)*

*Online: technical difficulty. The engagement is limited. More comfortable. We can do simple topics online. (J9)*

All residents favored the hybrid mode as a middle ground between the former and the later modes. The benefits of physical attendance are mostly recovered while reserving the online mode for more light topics, departmental mortality and morbidity discussion, and MCQs/SOE practice sessions.

*Hybrid: This is the best way; you have the benefit of having 1 day to have more time to study and you have the benefit to socialize with people and approach seniors directly. (J11)*

**Figure 2:**
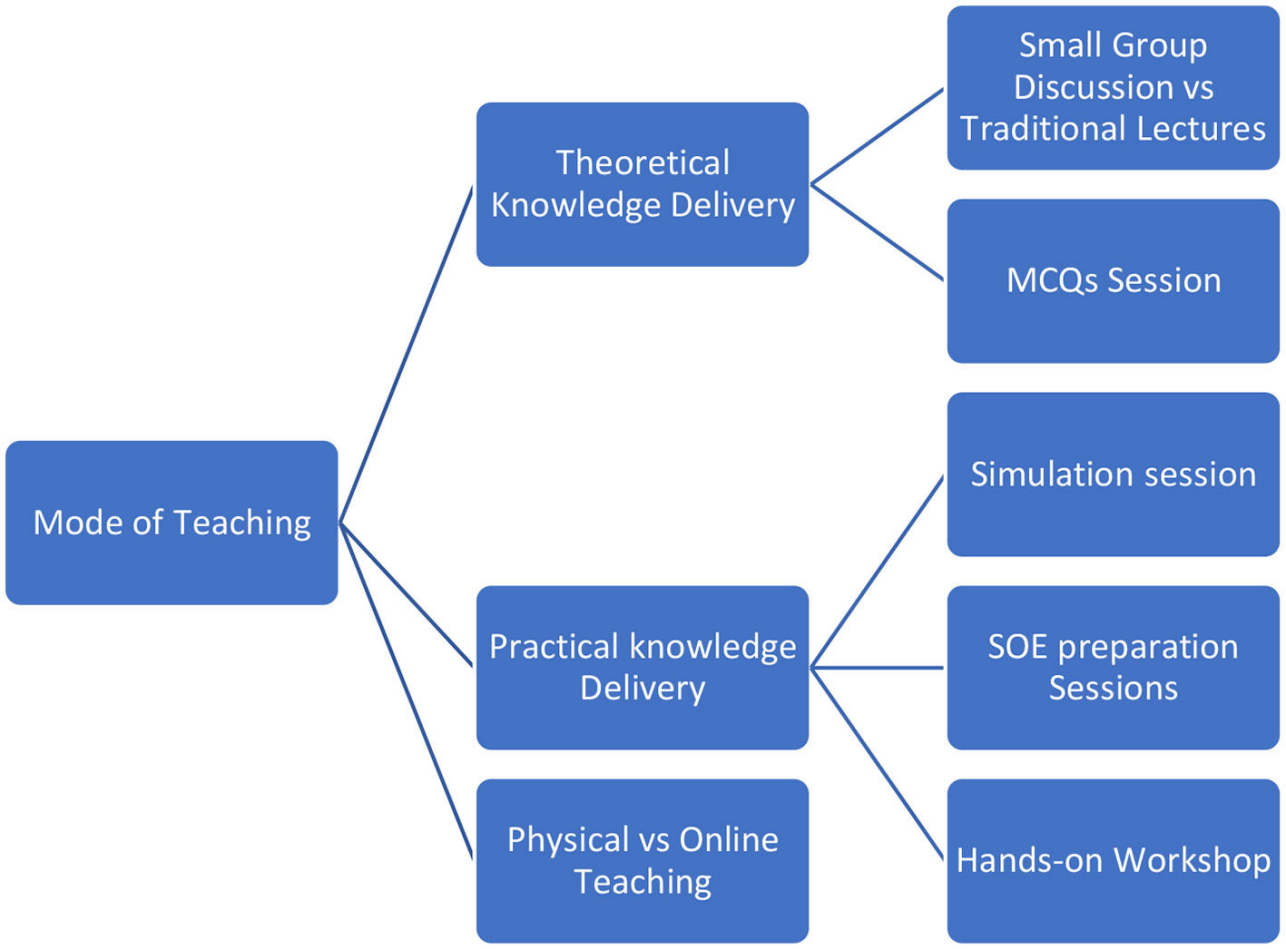
Sub-thematic analysis of Theme 1. Modes of teaching delivered to the residents.

Modes of teaching delivered to the residents are illustrated as a sub-thematic analysis of Theme 1 in Figure 2.

#### Theme 2: The learning environment

Residents were asked about their perceptions of the learning environment, and all of them found it friendly and family-like. The freedom to ask questions and share information without intimidation was highly spoken of by both juniors and seniors. Juniors precisely emphasized their willingness to commit mistakes in small group discussions.

*Big difference especially junior residents will not be afraid to make mistakes as opposed to making mistakes in front of the whole group. (J1)*

Residents also highlighted the positive attitude of the supervising consultants whom they had the chance to meet and share opinions with.

*The learning environment has zero blame and there's friendly consultant attitude. (S5)*

On the other hand, two senior residents noticed that anxiety about answering the presenter's questions was higher when the small group discussion was over, and the groups were called out to answer.

*In the small group, it was very safe. When we took it out, I felt the group uncomfortable to be called out randomly. Better for them to go on turns. [sic]*

**Figure 3:**
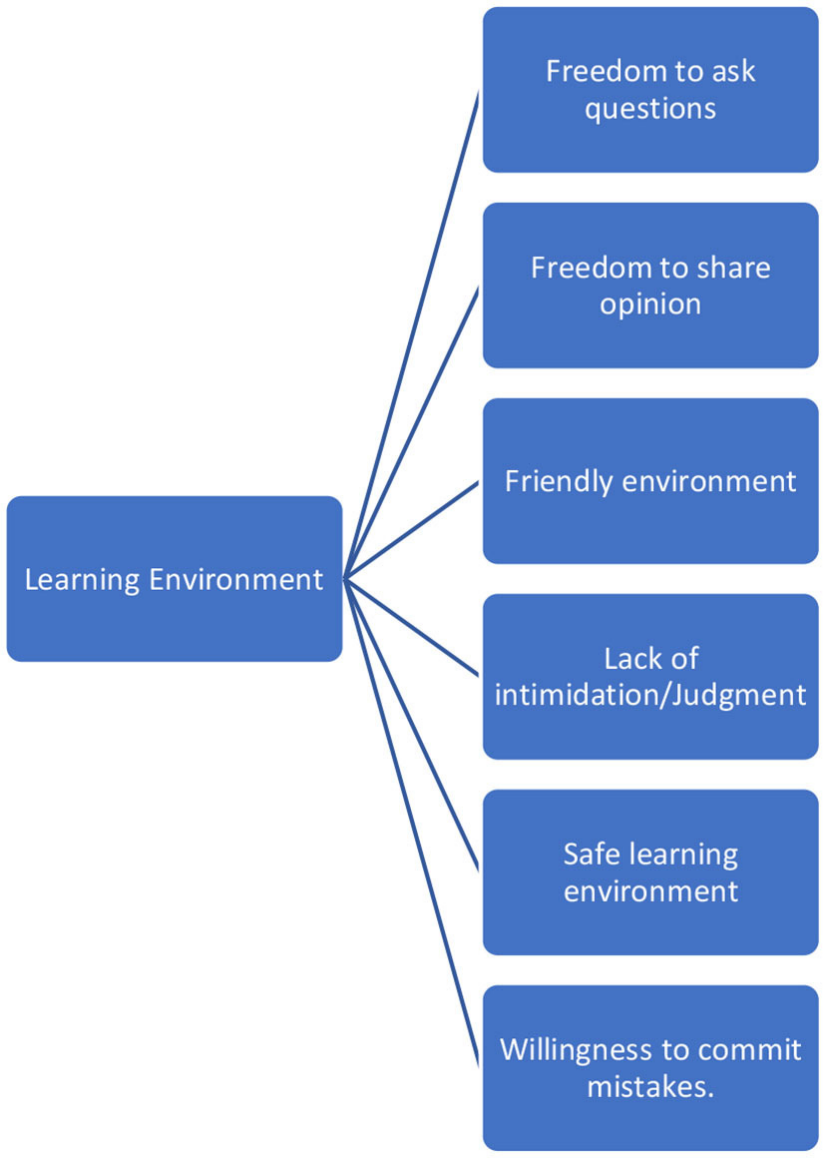
Sub-thematic analysis of Theme 2. Residents' point of view regarding the new learning environment.

The residents' point of view regarding the new learning environment is illustrated in Figure 3.

#### Theme 3: The impact of a modified educational approach

The impact of the modified educational approach on residents was explored in multiple aspects to measure its benefits. Residents highlighted the positive effect of the small group discussion on their level of topic understanding and knowledge retention. Moreover, they discussed its favorable impact on their home study schedule, their daily practice, exam preparation, and their confidence level before examinations.

The new approach helped them understand the topic and retain it longer through peer teaching and knowledge sharing compared to traditional lectures. The residents constantly spoke highly of the group's heterogeneity, where multiple residency levels contributed to the discussion, which made it more enriching. Moreover, juniors considered seniors in the group as a source of information, whereas seniors considered teaching their junior colleagues as a tool to help achieve a deeper understanding of knowledge.

*It increased the level of understanding on a junior level. Because I'm having 2 seniors in the group. I remember the seniors showing me how to study the topics. It makes me oriented more to the material. (J11)*

*The group has a bigger chance to deliver the point and explain difficult one. The knowledge delivery wasn't one way form the presenter only but from the group members as well. (J10)*

*It did improve the level of understanding. When you are the senior you'll need to explain things more to the junior which improved the level of understanding. (S6)[sic]*

However, two residents considered the repetitive pausing to discuss only one question disruptive to the flow of information. They wished to discuss more questions per time given for better time management and information processing.

*Frequent interruption to discuss after each question. And more time should be given with more questions to do and less interruption to the group study. (S1)*

Moreover, they considered studying the topic at home, followed by active discussion, and then revision at home, an example of a spaced repetition modality that helped with their knowledge retention and recall. In comparison to traditional lectures, their retention was better and longer. Apart from the motivation to read, most of the residents had aligned their home study schedule with the educational activity schedule; hence, they got better time management and less frustration about the number of topics they needed to read before the activity.

*Interactions coded the visual, auditory, and kinesthetic information better which helped retention. (J6)*

*If you don't align the study, you'll not get benefit. I used to align my home study with the academic activity to have the most benefit. After the academic the reading is considered as a revision. I felt a lot of benefit. (S6)[SIC]*

*In the Lecture based style, I didn't feel excited to read anything before the activity. (S10)*

On the other hand, those who did not align their home study schedule with the educational activity schedule perceived the pre-activity readings as a burden and additional work. Failure to align was either because the resident had different objectives, had less awareness of the benefits of alignment, or had excellent time management to read both their schedule and the educational activity schedule.

*I regret that I didn't align my study with the academic activity because we are covering everything in an amazing way. (J11)*

Most residents felt that the modified educational activity had impacted their daily practice in many ways, mainly by helping them develop a better approach to patients and decision-making. Debates and sharing knowledge around points of controversy gave them the confidence to face ambiguous cases. Others found connecting theory to practice easier, as discussions at the educational activity were often reflected in the clinical work. Many residents highlighted that simulation had positively empowered their leadership and communication skills during clinical work. Seniors constantly reported easier and better communication with juniors during the clinical shifts because they already spoke the same language and had developed the appropriate level of trust. However, one resident felt the educational activity had not impacted her clinical practice.

*The clinical practice improved because we were debating and discussing so you figure out the best approach. (J10)*

*I strengthened my relationship with my junior colleagues. We knew each other's level or approaches. Working with them was easier. I knew my juniors' weaknesses, so I took the opportunity in the shift to improve them. (S5)*

The most frequently mentioned impact of the modified educational approach on exam preparation was the alignment between the teaching and the assessment tools. Residents continuously perceived their extensive SOE preparation sessions as an effective tool to familiarize them with the end-of-year final SOE. Moreover, they could recall information mentioned in the educational activity through small group discussions and large group debates. As for the monthly MCQ sessions, many found them challenging and tricky, but up-to-date and educational. Some residents explained how the MCQ sessions motivated them to read and refer to more MCQ banks to prepare for the final promotion exam. Others elaborated on the positive effect of assimilating the exam environment monthly on the final year exam.

*During the oral, the SOE sessions were very helpful. We were prepared and I was thankful. Other centers noticed that were are good are the oral exam preparation because of the monthly sessions. (J11)*

*-MCQs were educational but tricky unlike the board exam. The more MCQs you do the better you are trained. (S7)*

When asked about their confidence level toward sitting for the final exam, all residents mentioned the boost in their confidence for the final OSCE exam compared to previous years, which helped lessen the anxiety that usually accompanies the final written promotion exam. Moreover, all residents obtained better marks by passing the final year promotion exam. Out of the 24 interviewed residents, 2 have not found a noticeable impact of the modified educational approach on exam preparation.

One of the constantly mentioned positive impacts is their attempts to adapt to the new style where audience engagement is mandatory. Residents who were not used to engaging their audience had to develop skills in thinking of the right questions to ask in order to deliver their knowledge. Adhering to the rule of slide presentation made their slides better with fewer written words and more pictures; hence, it was more entertaining. Residents also mentioned that anxiety was lowered when presenting because they knew the responsibility of knowledge delivery was mutual between them and the audience. One of the critical skills the residents mentioned was time management while presenting. Staying focused between the several pauses for discussion and the need to finish on time was initially difficult but then it got easier with time and practice.

*I developed the skill of how to make the lecture for the discussion, how to manage the time, how to covert the lecture to discussion, at first it was a distaste later it was piece of cake. (J11)*

Some residents also mentioned increased confidence when presenting in front of a large group and developing group management skills.

One of the significant perceptions noticed among the residents was the strong relationship they developed along the way with each other. Juniors looked up to their seniors with gratitude and respect, while seniors looked after their junior colleagues with much attention and care.

*I gained their trust (referring to the. juniors). They came for study advice I became a mentor for them. (S2)*

**Figure 4:**
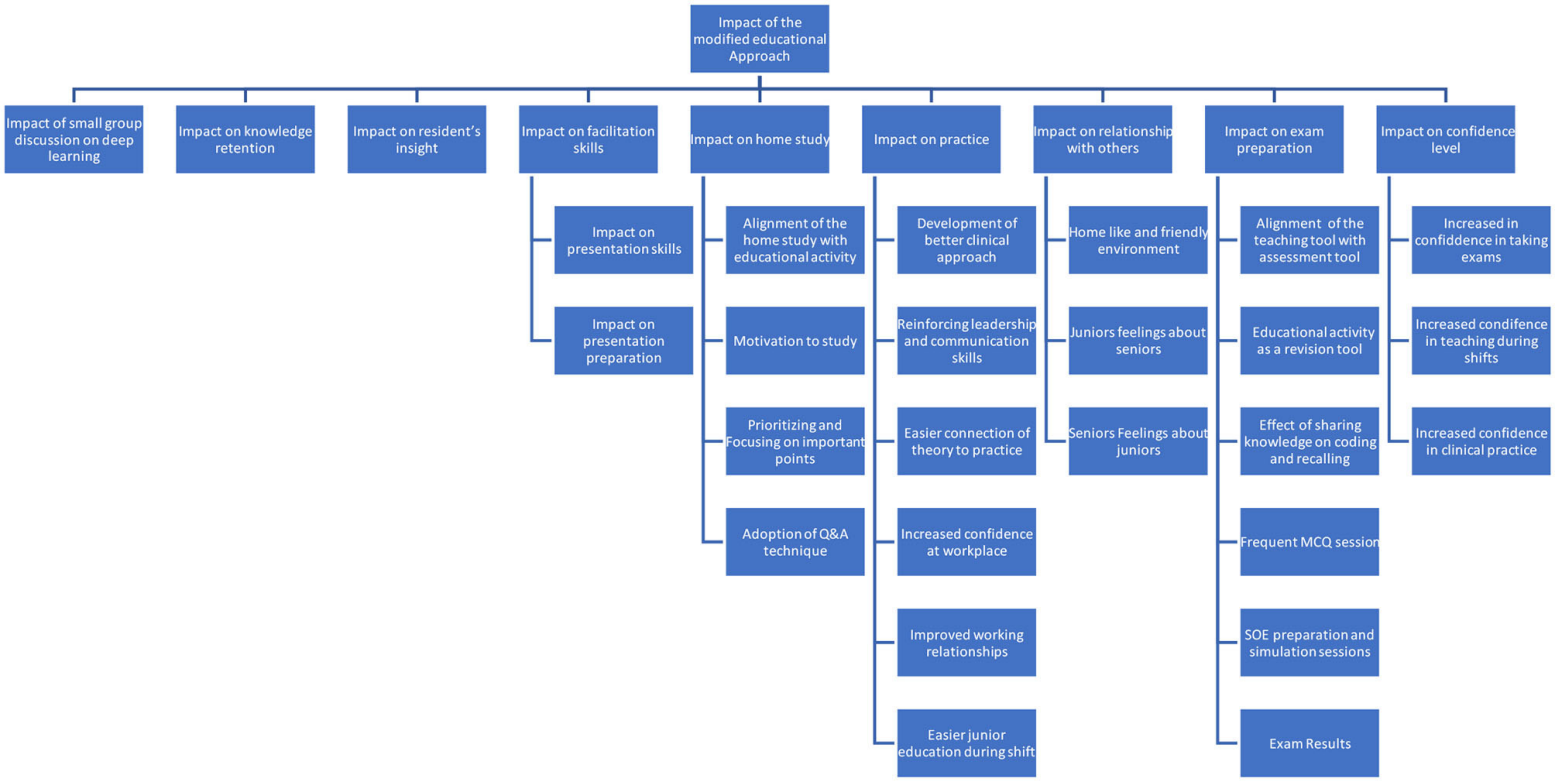
Sub-thematic analysis of Theme 3. Impact of the modified educational approach on different learning and social aspects as mentioned by residents.

The impact of the modified educational approach on different learning and social aspects as mentioned by residents is illustrated in Figure 4.

#### Theme 4: Challenges

The perception of challenges that the residents experienced along the way was crucial to properly putting together and improving plans for the next academic year. Acceptance of change was one of the mentioned challenges. Most residents had difficulty accepting the new modification in their learning experience, despite dissatisfaction with how educational activities used to be. It took them a while to experience the benefits of the modified approach to submit to these changes.

*I had a challenge to accept the change. But when we agreed that now we know better we got convinced. (S1)*

Residents took 4–9 weeks to adapt to the modified way of teaching. Adaptation varied depending on their personality type and residency level. Juniors were faster to adapt than seniors, especially the new residents who did not have prior experience with the educational activity's older versions. Resistance to change and longer adaptation time were most noticed among residency levels 2 and 3. Level 4 residents were more ready to accommodate the changes and accept the responsibility.

*- Different characters of residents and how deal with everyone was a challenge because you have to know all of them because you will work with them. Dividing us to small groups made the adaptation to the large number easier. (J3)[SIC]*

R1 needed help adapting to the enormous amount of curriculum they must cover. Some residents had difficulty adapting to speaking up and sharing their knowledge and opinions, and others had to take some time to adapt to the different personalities in the group. Moreover, a few junior residents needed help adapting to the new way of presentation.

*-We had to speak and interact rather than listening against my introvert personality. Adaptation took some time approximately 4 weeks. (J6)[SIC]*

One of the challenges the residents encountered was pre-reading the topics before attending the educational activity in order to participate and collaborate in the discussion. Many residents, especially the R1s, complained about the knowledge they needed to cover. Others acted differently and read only the objectives. The less complaining residents were those who managed to align their home study schedule with the educational activity schedule.

*The topics were huge to us and big struggle because we didn't have previous exposure. But I was assured that I will know this knowledge by time. (J9)[SIC]*

*I need to read all topics before I come because I'm the senior. The long time was exhausting. (S3)*

Another huge challenge for most residents was the amount of mental effort they needed to exert in 6 h compared to the traditional way of teaching. Many residents attributed the mental exhaustion to the continuous attention they needed to pay during the discussion and their active participation. Some residents highlighted that they could reach the end of the activity cognitively saturated with energy loss to continue the afternoon part and accordingly gain less benefit. Other challenges were the early timing of the activity and the length of the day, especially for the group assigned to the simulation that week.

*Mental effort exerted during the activity was a challenge. Because we have to be ready to answer all the time. (J8)*

**Figure 5:**
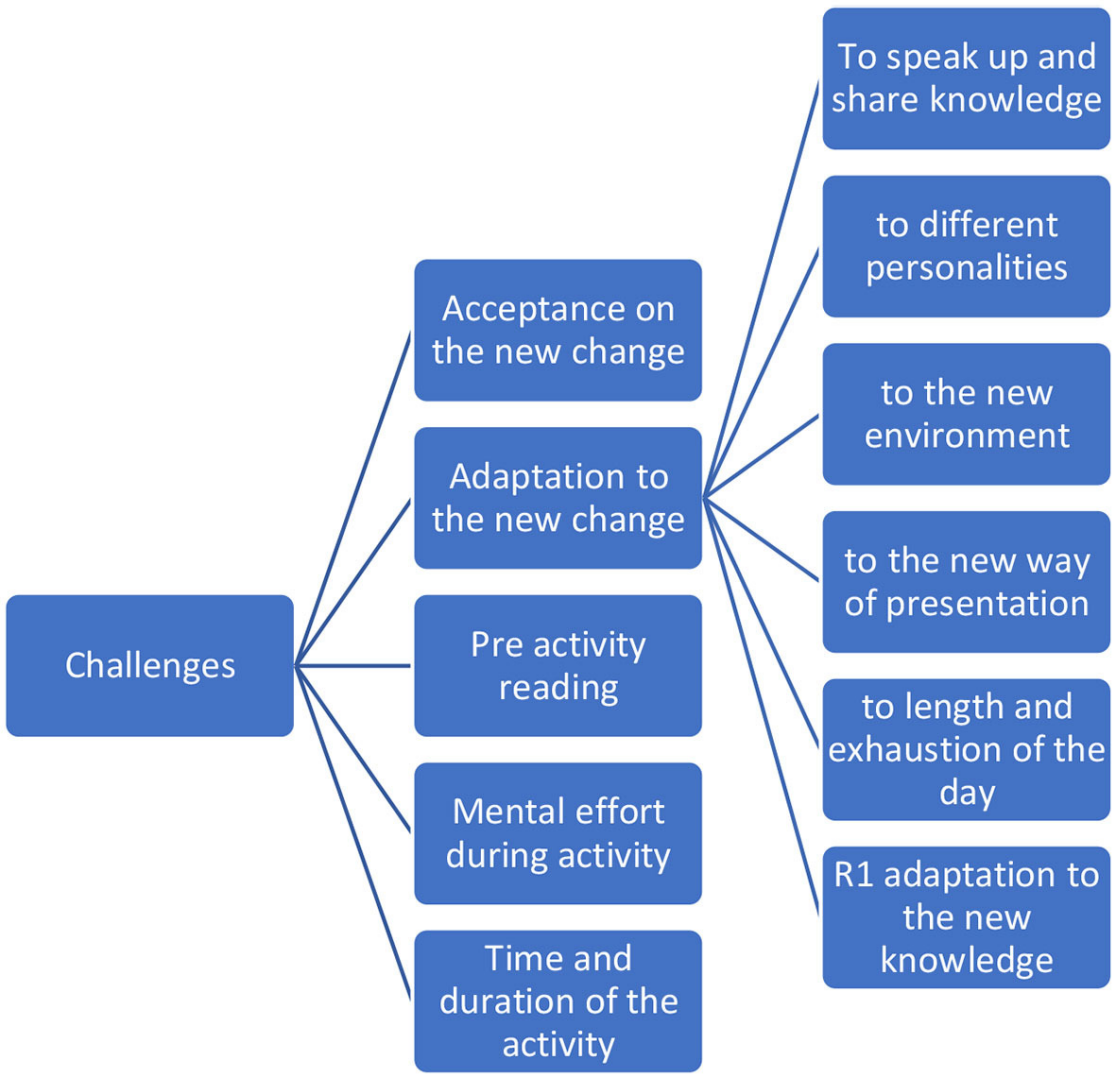
Sub-thematic analysis of Theme 4. Challenges toward the modified educational approach as perceived by residents.

The challenges toward the modified educational approach as perceived by residents are illustrated in Figure 5.

#### Theme 5: Satisfaction level

The high satisfaction level was almost consistent among junior residents but decreased slightly among seniors. General satisfaction included their engagement during the group discussions, better comprehension of the materials and longer retention, enthusiastic debates, peer teaching, and increased confidence to share knowledge.

Other elements were mainly about the friendly atmosphere, utilization of simulation, hands-on workshops, and SOE preparation sessions.

On the other hand, dissatisfaction elements were different for juniors and seniors. Juniors spoke mainly about the colossal curriculum they needed to cover, how the year started with tough topics for their level, and the self-study topics they wished they could discuss.

Seniors spoke about the basic level of discussion for specific topics, the lack of certain non-medical topics such as ethics and professionalism, the lack of consultants' lectures, and the more attention given to the junior level compared to the senior level.

Both levels, juniors and seniors, complained about the heaviness and length of the educational day, the limited benefit they perceived when group members did not share responsibility and read the topics beforehand, and the fact that we had to switch to complete online mode toward the middle of the year. Areas of satisfaction and dissatisfaction toward the modified educational approach as mentioned by residents are shown in Table 3.

**Table 3:** Sub-thematic analysis of Theme 5.

**Satisfaction level**	**Areas of satisfaction**	**Areas of dissatisfaction**
	A-Group-based discussion: 1-Engagement in the presented topics 2-Understanding the presentation material 3-Correction of misconceptions 4-Heterogenicity of the group members 5-Discussion with consultants 6-Easy applicability to practice 7-Excitement to read 8-Increased confidence to share knowledge B-No. of simulation sessions C-Amount and variety of topics covered D-Presence of objectives in each topic. E-Availability of feedback F-Friendly atmosphere G-SOE preparation session H-Monthly MCQ sessions	A-To juniors: 1-The heaviness of the topics at the beginning of the year 2-Some self-study topics best to be discussed B-To Seniors: 1-Basic levels of certain topics 2-Lack of non-medical topics 3-Lack of consultants' lectures 4-Less benefit when members do not engage C-To both levels: 1-Length and heaviness of activity 2-Less benefit when the topic is not previously prepared 3-Online mode

Areas of satisfaction and dissatisfaction toward the modified educational approach as mentioned by residents.

## Discussion

The aim of the study was to explore the residents' experiences regarding implementing the new teaching and learning approach. The teaching strategies used in the new educational activity were based on multiple learning theories, including constructivism (22) and social learning theory (23). Moreover, simulation was heavily incorporated into the educational activity, allowing for practical training of the learned theory and consolidation of that knowledge using Kolb's experiential learning [Theory (24)].

Inquiry-based learning, case-based learning, and small group learning are examples of collaborative learning strategies used to enrich educational activities' quality, increase resident satisfaction, increase knowledge retention, and enhance resident engagement. One of the principles of Adult Learning Theory is that adults are self-motivated and promote their learning process (25). Thus, the Educational Activity Committee (EAC) of the training program mentioned earlier was formed by residents of different levels and chaired by the program director. The committee worked closely on reforming the teaching strategies and didactic content of the curriculum.

The new modified teaching approach faced the challenges of limited human, physical, and financial resources. In terms of human resources, educational activity used to rely heavily on consultants' involvement in terms of knowledge delivery. With the program separation and limited consultants' availability, the new approach considered all final-year residents as a source of excellent knowledge and experience for the rest of the group. Accordingly, each group had heterogeneous resident levels, allowing for hierarchical knowledge sharing. On the other hand, the consultants' role was diverted to supervision and moderation of the discussion. Such empowerment for the residents gave them more room to own their learning and engage in scientific debates to answer their inquiries instead of the ancient perception of having the consultant as the only source of information.

Although the general perception of residents toward the change was considered very good, a few points had to be explored for better satisfaction and a durable impact. Active learning is considered a powerful tool that integrates theory into practice (26). With such integration, one can ensure effective residency training. For years, the residents in this study felt disengaged from the educational activity and perceived it as a burden with no noticeable impact. Students' engagement is crucial for better performance and a more enjoyable learning journey (27). Several factors influence students' engagement in higher education, including age, gender, socioeconomic status, behavior, academic disciplines, institutional characteristics, perceived workload, and relationships with peers and teachers (28). The modified educational approach explored in this study has worked on several factors to achieve engagement and better learning. Creating the proper environment for learning had a powerful impact on the success of such an approach, which empowered the active learning process. There are three attributes to ensure a safe environment in simulation psychologically: the ability to commit mistakes without judgment, the facilitator qualities, and the foundational activities such as the presence of objectives, preparation, and orientation (29). These factors were replicated in the modified educational approach to create a safety net where residents can freely learn. Furthermore, the positive impact of the modified approach on the relationship between residents was reflected in their workplace, where they shared knowledge, trust, and motivation to educate and learn.

Peer teaching is any educational activity involving students engaging in the educational cycle as partners (30). There are numerous studies favoring peer teaching and enumerating its advantages. The benefits include improved self-responsibility, communication skills, motivation to teach or learn, and critical thinking (30). In the modified educational approach, peer teaching was integrated and developed through the heterogenicity of the group members. Not only did seniors share their knowledge and experience with juniors, but they also developed mentoring and coaching skills with their team members. Juniors highly value such seniors' behavior.

One of the most significant advantages of the modified educational approach was its higher retention compared to traditional lectures. From the work of Craig Johnson and his group, organization, elaboration, mental cueing, and evaluation have contributed to designing frameworks that would aid in better memorization regarding group discussion (31). Apart from the discussion that helps encode the information better, residents highlighted the spaced repetition technique. *Spaced repetition* is a learning method that allows students to learn more in less time. Although discovered over a decade ago, the technique has an enhanced effect on meaningful retention (32) and is widely used among school students (33), medical schools (34), and other sciences (35). The modified educational approach served as a spaced repetition tool as residents had to study the material before the educational activity, discuss the material in the educational activity, and review the topic afterward. Such techniques result in more long-term learning compared to mass information (36).

One of the principles of curriculum design is constructive alignment (37). According to Biggs, constructive alignment of the intended learning outcomes, educational tools, educational activities, and assessment tools is warranted to achieve students' deep learning. It has been a constant complaint by residents in this study that studying for the educational activity does not meet their goal of studying for the end of year exam. Thus, they needed to have a separate study track to prepare for their end-of-year promotion exam. With its collective theoretical and practical activity components, the modified educational approach successfully prepared residents for their final written promotion and structured oral exams. Since the feedback was integrated after each activity, evidence of the resident's achievement of the intended learning outcome was assured (38). Moreover, residents who aligned their home study schedule with their academic schedule had smoother learning journeys and more benefits.

It is hard to compare students' achievements in the end-of-year exam conducted last year and the years before since different cofounders exited with different contributing factors between the 2 years. In this study, the boost in confidence level prior to the SOE would be a significant finding since it was among all residents interviewed. Confidence levels prior to the written promotion exam had not changed much, but their anxiety toward sitting for the exam had improved. Moreover, the pass rate was 100% in both written exams and SOE, unlike the year before.

Another important highlight of the study is the impact of the modified educational approach on the residents' facilitation skills. According to Hogan, facilitators walk participants through different appropriately chosen processes to reach their learning goals (39). Unlike the traditional lecture, which is mainly a teacher-centered process, facilitation makes the students the center of the educational cycle (40). According to Alias Masek, teaching experience had no impact on the capability of the facilitators; hence, facilitation skill development was observed among all residency levels. Out of the three facilitators' modes, hierarchy, autonomy, and cooperation, autonomy and cooperation were the most noted facilitation modes among the residents. They monitored the task, facilitated the dialogue between members, and led the discussion.

Conceptual change theories have been a hot topic to discuss for decades. One of the oldest theories belonged to Posner et al. (41) who proposed a framework necessary for conceptual change: dissatisfaction with the old conception, new conception intelligibility, plausibility, and fruitfulness. Most of the residents in this study were ready to accommodate and adapt to the new changes since they were already dissatisfied with the old way of teaching. By the end of the year, changes made sense to them and were fruitful, as evidenced by the modified approach's satisfaction and positive impact on various aspects. They also listed the advantages of the new modified approach and the disadvantages of the previous practice, which helped with their comprehensive understanding of the new change.

Despite their accommodation of the new change, several challenges emerged along the way. One of the elaborated-on challenges was the amount of cognitive effort residents had to exert in each educational activity. Moving from the sedentary spectrum, where they used to be passive learners, to the spectrum of active learning made them to be in a constant state of attention. The process was mitigated by frequent breaks. Moreover, their agreement to combine the online teaching mode and the physical attendance mode opened a new perspective as part of the solution.

The vast curriculum the residents needed to cover was another culprit, which was slightly lessened by assigning some topics for self-study which they could cover in their own time and at their own pace. Nevertheless, the importance of formulating study groups at home has always been highly encouraged to overcome such a factor.

The time that it took for the residents to accept the change was brief since they were already unsatisfied with the old method. But another discussed challenge was the time they took to adapt to the change, especially being in one group where everyone was expected to share knowledge and participate. They took time to adapt to each other's different personalities, find out about each other's strengths and weaknesses, and develop appropriate ways to communicate effectively. Bridgeland et al. believes developing competencies that include self-management and awareness, social awareness, skills to interact within a group, and decision-making is critical for students' success (42). Another adaptation period was required to get used to reading the material before the educational activity and adapting to the new presentation style.

The perception of satisfaction depends on multiple factors, including age, gender, socioeconomic status, and students' personality types (28). Another study looked at more factors, including the teachers' expertise, learning environment, courses provided, and classroom facilities (43). An overall sense of satisfaction was obtained from all interviewed residents. More satisfaction was noted in R1, R2, and R4, where the minor level was R3. The R1 and most of the R2 experienced the advantage of peer teaching, which added to their satisfaction level. R4 was already preparing for the final exam and was more satisfied with the broad curriculum coverage. R3 had the lowest satisfaction level.

Many recommendations were suggested to improve the current style. Integrating such recommendations will lead to more successful implementation and more benefits and satisfaction. One of the recommendations that would allow for less mental exhaustion is to cover some topics with a case-based discussion style that does not involve grouping and pausing to discuss answers to the presenter's questions. Close monitoring of the presentations' time and quality will efficiently remove the possibility of staying beyond expected hours. Presentation quality can be monitored by reinforcing that all presentations should be discussed with mentors.

Some residents suggested changing the groups frequently for junior residents to get more exposure to different seniors. Considering the time it takes to formulate strong bonds between group members, groups might be given a chance to change twice a year.

Suggestions to change the time of the MCQ sessions to be the last activity of the block are logical suggestions. Other suggestions included increasing the number of consultant lectures and integrating more updates and controversies to satisfy seniors. Including non-clinical topics in the educational activity was also suggested. All suggestions were integrated into this academic year's educational activity, striving for better outcomes.

## Strengths and limitations

According to the best of our knowledge, this is the first qualitative study that addresses changing the pattern of education in the emergency medicine program from passive to active learning. Another strength is the successful implementation of the small group discussion with a large group of residents. An additional strength is that the study addresses a holistic approach to educational activity, which used to be theoretical with few workshops. Constructive alignment of the curriculum was a substantial addition that needed to be explored. The approach is excellent for programs that have limited faculty resources. To allow replication, an educational intervention must be presented in a clear and descriptive manner. Therefore, the intervention in this article followed the criteria suggested by a systematic review led by Meinema et al. (44).

Another strength is the availability of physical resources. The modified approach relied heavily on the physical resources of the College of Medicine-Jeddah (COM-J) at King Saud bin Abdulaziz University for Health Sciences (KSAU-HS). Resources included the lecture hall, technical support, audio-visual support, and simulation center support. As for the financial support, the activity was self-funded by the residents who sponsored the catering and the appreciation events. Replicating the process with limited physical resources would take a lot of work. Good planning and preparation can mitigate the situation by considering factors such as utility and practicality.

This qualitative study only captured the self-perceived views of the participants regarding the new approach. Moreover, there might be a potential unintended influence by the interviewer on the interviewee's responses regarding their satisfaction since the interviewer is the program director. However, the influence was limited since both parties were keen to explore the experience for future improvement.

## Conclusion and recommendation

This study highlights the benefits and challenges of incorporating active learning into the postgraduate emergency medicine residency training program. Moreover, it links the benefits directly to the residents' home study, work environment, clinical practice, and exam preparation. It also shows improved self-perceived satisfaction compared to passive learning. The study tested several modes of information delivery with a detailed explanation of each one's advantages and disadvantages and concluded that mixing both modes, online and physical attendance, is highly regarded by the residents.

The authors strongly recommend changing the learning culture from passive to active teaching in all post-graduate residency training programs. Moreover, the authors recommend mandatory curriculum alignment of the educational tools in the weekly educational activity to the assessment tools at the end of the year for better residents' exam preparation, confidence level, and hopefully better exam performance.

## Data availability statement

The raw data supporting the conclusions of this article will be made available by the authors, without undue reservation.

## Ethics statement

The studies involving human participants were reviewed and approved by King Abdullah International Medical Research Center, Riyadh, Saudi Arabia. The patients/participants provided their written informed consent to participate in this study.

## Author contributions

KB: interviewing candidates, recording interviews, records transcription, transcripts thematic analysis, and writing manuscript. SA-N and AA: reviewing transcription, reviewing thematic analysis, and reviewing manuscript. All authors contributed to the article and approved the submitted version.

## References

[1] Freeman S Eddy SL McDonough M Smith MK Okoroafor N Jordt H. Active learning increases student performance in science, engineering, and mathematics. Proc Nat Acad Sci. (2014) 111:8410–5. 10.1073/pnas.1319030111 24821756 PMC4060654

[2] Cashin WE. Improving lectures. Idea Paper No. (1985) 14:3.

[3] Alaagib NA Musa OA Saeed AM. Comparison of the effectiveness of lectures based on problems and traditional lectures in physiology teaching in Sudan. BMC Med Educ. (2019) 19:1–8. 10.1186/s12909-019-1799-0 31547817 PMC6757398

[4] Brown G Manogue M. AMEE medical education guide No. 22: refreshing lecturing: a guide for lecturers. Med Teach. (2001) 23:231–44. 10.1080/01421590120043000 12098394

[5] Bonwell CC Eison JA. Active Learning: Creating Excitement in the Classroom. ASHEERIC Higher Education Report No. 1. Washington, D.C: George Washington University (1991).

[6] Laal M Laal M. Collaborative learning: what is it? Proc Soc Behav Sci. (2012) 31:491–5. 10.1016/j.sbspro.2011.12.092

[7] Johnson DW Johnson RT Smith KA. Cooperative learning returns to college what evidence is there that it works? Change Mag Higher Learn. (1998) 30:26–35. 10.1080/00091389809602629

[8] Johnson DW Johnson RT Smith KA. Cooperative learning: improving university instruction by basing practice on validated theory. J Excell College Teach. (2014) 25:85–118.9488851

[9] Springer L Stanne ME Donovan SS. Effects of small-group learning on undergraduates in science, mathematics, engineering, and technology: a meta-analysis. Rev Edu Res. (1999) 69:21–51. 10.3102/00346543069001021

[10] Roseth CJ Johnson DW Johnson RT. Promoting early adolescents' achievement and peer relationships: the effects of cooperative, competitive, and individualistic goal structures. Psychol Bull. (2008) 134:223–46. 10.1037/0033-2909.134.2.223 18298270

[11] Slavin RE. When does cooperative learning increase student achievement? Psychol Bull. (1983) 94:429–45. 10.1037/0033-2909.94.3.429

[12] O'Donnell AM. The role of peers and group learning. in Handbook of Educational Psychology. P. H. E. A. Winne and A. Patricia (Eds.), Mahwah, NJ: Lawrence Erlbaum Associates Publishers (2006) (pp. 781–802).

[13] Van Blankenstein FM Dolmans DH van der Vleuten CP Schmidt HG. Which cognitive processes support learning during small-group discussion? The role of providing explanations and listening to others. Instructional Sci. (2011) 39:189–204. 10.1007/s11251-009-9124-7

[14] Deng F. Literature review of the flipped classroom. Theory Pract Lang Stud. (2019) 9:1350–6. 10.17507/tpls.0910.14

[15] Thistlethwaite JE Davies D Ekeocha S Kidd JM MacDougall C Matthews P. The effectiveness of case-based learning in health professional education. A BEME systematic review: BEME Guide No. 23. Med Teach. (2012) 34:e421–44. 10.3109/0142159X.2012.680939 22578051

[16] McLean SF. Case-based learning and its application in medical and health-care fields: a review of worldwide literature. JMECD-S. (2016) 3:20377. 10.4137/JMECD.S20377 29349306 PMC5736264

[17] Berger G. Needs assessment lessons learned in Qatar: a flipped classroom approach. MedEdPublish. (2019) 8:48. 10.15694/mep.2019.000048.1 PMC10712580 38089336

[18] Suliman S Al-Mohammed A Al Mohanadi D Karim H Elbuzidi A Mubasher M. It is all about patients' stories: case-based learning in residents' education. Qatar Med J. (2020) 2019:17. 10.5339/qmj.2019.17 31903323 PMC6916428

[19] Barrow L. A brief history of inquiry-from dewey to standards. J Sci Teacher Educ. (2006) 17:265–78. 10.1007/s10972-006-9008-5

[20] Barron B Darling-Hammond L. Prospects and challenges for inquiry-based approaches to learning. Nat Learn Using Res Inspire Practice. (2010) 3:199–225. 10.1787/9789264086487-11-en

[21] Braun V Clarke V. Thematic analysis. Am Psychol Assoc. (2012) 32:17–37. 10.1037/13620-004

[22] Bodner GM. Constructivism: a theory of knowledge. J Chem Educ. (1986) 63:873. 10.1021/ed063p873

[23] Bandura A Walters RH. Social Learning Theory, Vol. 1. Prentice Hall: Englewood Cliffs (1977).

[24] Kolb DA. The process of experiential learning. Exp Learn Exp Source Learn Develop. (1984) 4:20–38.

[25] Knowles MS. Andragogy: adult learning theory in perspective. Commun College Rev. (1978) 5:9–20.

[26] Wrenn J Wrenn B. Enhancing learning by integrating theory and practice. Int J Teaching Learn High Edu. (2009) 21:258–65.

[27] Denovan A Dagnall N Macaskill A Papageorgiou K. Future time perspective, positive emotions and student engagement: a longitudinal study. Stud Higher Edu. (2020) 45:1533–46. 10.1080/03075079.2019.161616832416543

[28] Tani M Gheith MH Papaluca O. Drivers of student engagement in higher education: a behavioral reasoning theory perspective. Higher Education. (2021) 82:499–518. 10.1007/s10734-020-00647-7

[29] Turner S Harder N. Psychological safe environment: a concept analysis. Clin Simulat Nurs. (2018) 18:47–55. 10.1016/j.ecns.2018.02.004

[30] Stigmar M. Peer-to-peer teaching in higher education: a critical literature review. Ment Tutor Partnership Learn. (2016) 24:124–36. 10.1080/13611267.2016.117896334785209

[31] Johnson C Gadon O Carlson D Southwick S Faith M Chalfin J. Self-reference and group membership: evidence for a group-reference effect. Eur J Soc Psychol. (2002) 32:261–74. 10.1002/ejsp.8332705946

[32] Ausubel DP Youssef M. The effect of spaced repetition on meaningful retention. J Gen Psychol. (1965) 73:147–50. 10.1080/00221309.1965.9711263 14316956

[33] Yeh MKC Toshtzar A Guertin L Yan Y. Using spaced repetition and gamification to enhance K-12 student science literacy with on-demand mobile short reads. in 2016 IEEE Frontiers in Education Conference (FIE). IEEE (2016). p. 1–4.

[34] Augustin M. How to learn effectively in medical school: test yourself, learn actively, and repeat in intervals. Yale J Biol Med. (2014) 87:207. 24910566 PMC4031794

[35] Teninbaum GH. Spaced repetition: a method for learning more law in less time. J High Tech L. (2016) 17:273.

[36] Kang SH. Spaced repetition promotes efficient and effective learning: policy implications for instruction. Policy Insights Behav Brain Sci. (2016) 3:12–9. 10.1177/2372732215624708

[37] Biggs J. What the student does: teaching for enhanced learning. Higher Edu Res Develop. (1999) 18:57–75. 10.1080/0729436990180105

[38] Ali L. The design of curriculum, assessment and evaluation in higher education with constructive alignment. J Edu e-Learn Res. (2018) 5:72–8. 10.20448/journal.509.2018.51.72.78

[39] Hogan C. Understanding Facilitation: Theory and Principle. London: Kogan Page Publishers (2005).

[40] Masek A. Mode and dimension of facilitation in student-centred learning approach: a comparison of teaching experience. Int J Active Learn. (2019) 4:24–32.

[41] Posner GJ Strike KA Hewson PW Gertzog WA. Accommodation of scientific conception: towards a theory of conceptual change. Sci. Edu. (1982) 66:211. 10.1002/sce.3730660207

[42] Bridgeland J Bruce M Hariharan A. The Missing Piece: A National Teacher Survey on How Social and Emotional Learning Can Empower Children and Transform Schools. A Report for CASEL. Civic Enterprises (2013).

[43] Butt BZ Ur Rehman K. A study examining the students satisfaction in higher education. Procedia-Soc Behav Sci. (2010) 2:5446–50. 10.1016/j.sbspro.2010.03.888

[44] Meinema JG Buwalda N van Etten-Jamaludin FS Visser MR van Dijk N. Intervention descriptions in medical education: what can be improved? A systematic review and checklist. Acad Med. (2019) 94:281. 10.1097/ACM.0000000000002428 30157087 PMC6365274

